# Looking back and planning ahead: the tobacco endgame and controlling tobacco industry interference in Aotearoa New Zealand

**DOI:** 10.1080/03036758.2025.2455498

**Published:** 2025-04-10

**Authors:** Janet Hoek, Andrew Waa, Richard Edwards, Melissa-Jade Gregan, Anna Graham-DeMello

**Affiliations:** a Department of Public Health, Ōtākou Whakaihu Waka Pōneke | University of Otago, Wellington, Aotearoa New Zealand; b Rōpū Rangahau Hauora a Eru Pōmare, Department of Public Health, Ōtākou Whakaihu Waka Pōneke | University of Otago, Wellington, Aotearoa New Zealand

**Keywords:** Tobacco industry interference, tobacco endgame, smokefree policy, ‌Policy Dystopia Model, coroporate lobbying‌

## Abstract

Smoking causes at least 16 different cancers and has resulted in persistent health inequities. Aotearoa New Zealand was initially a Tupeka Kore (Tobacco Free) nation and Māori leaders have led calls for measures that would end the harms smoking imposes. Their vision inspired a national movement and later saw a new law introduced that mandated denicotinisation, reduced the supply of tobacco, and introduced a smokefree generation. The National-led coalition Government repealed these measures, drawing on arguments often advanced by tobacco companies. Using the Policy Dystopia Model, we analysed tobacco industry narratives and reviewed politicians’ use of industry arguments. We found coalition Government members drew on prohibition and illicit trade arguments, which segued into public safety concerns, despite weak evidence for these claims. To protect innovative public health policies, we argue for stronger lobbying regulation to reduce tobacco companies’ ability to interfere with policy making and improve compliance with Article 5.3 of the World Health Organization’s Framework Convention on Tobacco Control. Stronger regulation of lobbying would protect critical public health policies that could profoundly reduce cancer rates from commercial interests.

## Introduction

Measures that reduce smoking prevalence offer profound public health, social and economic benefits, and could greatly reduce cancer rates and inequities. In this article, we first summarise cancer-related harms caused by smoking in Aotearoa before exploring the Tupeka Kore (Tobacco Free) vision articulated by Māori leaders, which outlined a new approach (an ‘endgame’) to ending the tobacco epidemic. To understand this highly innovative approach, we explain Aotearoa’s tobacco policy history and analyse the movement from an individually focussed to a more systemic approach. We then examine the repeal of the endgame measures, analyse arguments advanced by tobacco companies, and consider how these may have influenced the coalition Government’s smokefree policy approach.

### Smoking, cancer and inequities

Evidence of smoking’s lethal effects has accrued for several decades. Early studies established smoking causes lung cancer (Doll and Hill 1950, 1954; Wynder and Graham 1950; Doll et al. 1994), and subsequent work found it causes more than 16 different cancers affecting multiple organs (Blakely et al. 2013; Carter et al. 2015; Cancer Research UK 2023; Quit Australia 2023). Every year, between 4500 and 5000 New Zealanders die from an illness caused by smoking; nearly one in four deaths (22.6%) among Māori are attributable to smoking (around one in eight deaths (12.3%) for non-Māori and non-Pacific people) (Health Coalition Aotearoa 2024), and smoking remains one of the leading causes of cancer and premature death in Aotearoa and internationally (Dai et al. 2022).

Lung cancer is arguably the most well-known cancer caused by smoking, with recent studies reporting that smoking causes more than 80% of lung cancers (Whiteman et al. 2015; Te Aho o te Kahu Cancer Control Agency 2021). Lung cancer incidence and mortality are high in Aotearoa New Zealand (Aotearoa) and this disease places a particular burden on Māori. In 2021 (the most recent year for which data are available), 2542 people living in Aotearoa were diagnosed with lung cancer (559 Māori) and, between 2017 and 2021, 9030 people died of lung cancer (1920 Māori) (Health New Zealand Te Whatu Ora 2024). Māori have a poorer survival rate for lung cancer and are 30% more likely to die than non-Māori diagnosed with lung cancer (Gurney, Robson, et al. 2020; Gurney, Stanley, et al. 2020). Pacific peoples living in Aotearoa also have a higher lung cancer incidence and a lower survival rate relative to European New Zealanders (Cleverley et al. 2023).

In 2022/2023, daily smoking prevalence among Māori was 17.1%, nearly three times the rate among European/Other (6.1%) (Ministry of Health 2023). Although absolute rates of smoking have declined from 36.3% in 2012/2013 among Māori and 13.5% among European/other, the relative difference has not changed during the last ten years and smoking rates continue to be around three times higher among Māori than European/other (Ministry of Health 2023).

Because the inequities that have led to higher smoking prevalence among Māori and poorer treatment outcomes reflect multiple factors within and beyond the health system, some researchers have called for a comprehensive response that addresses wider determinants of health (Hill et al. 2005; 2013; Davies et al. 2021). Indigenous scholars have also issued challenges to consider colonisation and racism, fundamental determinants of health (Curtis et al. 2023).

### Returning to a Tupeka Kore nation

Inequities in smoking prevalence and the harms that follow represent a bitter irony for Māori, who were a Tupeka Kore people prior to European contact. Following first contact, tobacco quickly became an article of trade and inducement that exploited Māori (Reid and Pouwhare 1991). Distribution of tobacco rations to troops during both World Wars saw Māori soldiers addicted to smoking when they returned from fighting. As smoking became normalised, prevalence rose and by the 1940s, more than 60% of Māori smoked (Reid and Pouwhare 1991; Easton 1995; Walsh and Wright 2020). Romanticised portrayals of Māori smoking, targeting by tobacco companies, and appropriation of Indigenous imagery also increased smoking prevalence among Māori (Te Reo Mārama 2006; Waa et al. 2020).

Yet despite consistent evidence that smoking prevalence was higher among Māori than among any other population group, health promotion campaigns did not initially recognise Māori as a unique group with specific interests and priorities. Thus while ‘mainstream’ interventions saw smoking prevalence fall, the large and persistent inequities between Māori and non-Māori did not decrease and even increased during some periods (Tautoko and Kore 2003; Ministry of Health 2023). For example, although smoking prevalence declined during the 1980s and 1990s, increasing socio-economic inequity and disadvantage experienced by Māori saw inequities in smoking prevalence increase (Barnett et al. 2005).

Māori leaders have long advocated centering understanding of tobacco-related harm within a Māori worldview and have raised concerns about the many health, social and financial inequities tobacco use imposes on their peoples (Tautoko and Kore 2003). In 2005, concerned that tobacco control initiatives had thus far failed to reduce these inequities, and frustrated at the lack of progress, they articulated a Tupeka Kore vision that aimed to return Aotearoa to its original tobacco-free status (The Māori Party 2006). Tupeka Kore quickly became a sector wide movement as other groups joined the call for stronger policy measures; in 2009, the wider tobacco control sector set a goal of achieving Tupeka Kore by 2020 (Smokefree Coalition 2009).

Māori political leaders also supported the Tupeka Kore movement and initiated the Māori Affairs Select Committee (MASC) Inquiry into the tobacco industry in Aotearoa and the consequences of tobacco use for Māori (Māori Affairs Committee 2010). This landmark investigation saw members travel throughout Aotearoa where they heard from whānau (family), community leaders, health practitioners and researchers; they also required tobacco companies to attend hui (meetings) and answer questions. The kōrero (discussion) informed a detailed report that contained several recommendations and called on the Government to adopt a goal of making Aotearoa smokefree by 2025 (Māori Affairs Committee 2010). The Government accepted this recommendation, thus formalising the Smokefree 2025 goal of ‘*reducing smoking prevalence and tobacco availability to minimal levels, thereby making New Zealand essentially a smoke-free nation by 2025’* (New Zealand Government 2011).

### The lean years: 2011–2020

Yet despite knowledge of factors that foster smoking uptake and complicate cessation (De Biasi and Dani 2011; Chaiton et al. 2016), no government introduced a co-ordinated strategy to realise the Smokefree 2025 goal. Instead, policy action was limited, piecemeal, and vulnerable to political vicissitudes (Wilson et al. 2011; Gendall et al. 2013; Witt et al. 2018). Mid-point smoking prevalence targets among Māori and Pacific peoples set by the MASC were not met (Ball et al. 2016) and relative inequities persisted (Ministry of Health 2023).

### The introduction of ‘endgame’ measures

The election of a majority Labour government and appointment of Dr Ayesha Verrall as Minister of Health in 2020 heralded a period of intense policy activity. Within a few months, Dr Verrall launched proposals for a Smokefree Aotearoa 2025 Action Plan (the ‘Action Plan’); her plan outlined a new and innovative approach to smokefree policy (Ministry of Health) that aimed to end rather than control the tobacco epidemic. Instead of addressing ‘demand-side’ factors and relying on individually-oriented cessation measures, the Action Plan acknowledged upstream determinants and addressed the addictiveness and widespread availability of tobacco products (New Zealand Government 2021).

Hailed as world-leading, subsequent legislation set out an endgame strategy that would lead smoking prevalence to fall rapidly among all population groups. Three evidence-based measures included a large reduction in tobacco availability, denicotinisation (to render tobacco essentially non-addictive), and a smokefree generation, which made the sale or supply of tobacco to anyone born on or after 01 January 2009 illegal (Ministry of Health 2021; New Zealand Government 2023). The plan specifically recognised commitments to Te Tiriti o Waitangi and to priority groups, and ensured Māori and Pacific peoples held leadership roles in decision-making.

Modelling suggested denicotinisation would have had the most rapid and profound effect on smoking prevalence (Ait Ouakrim et al. 2023). This measure also responded to the widespread regret people who smoke experience and the difficulty many have in becoming smokefree (Wilson et al. 2009; Edwards, Hoek, Waa, et al. 2022b). The retail reduction and smokefree generation measures reframed tobacco and asserted young people’s rights to protection from an innately harmful product; these latter policies were expected to have medium to longer-term impacts on smoking prevalence (Berrick 2013; Pearson et al. 2015; van der Deen et al. 2018). Overall, the policies recognised that smoked tobacco, a product that will kill two-thirds of its long-term users, should no longer be viewed and regulated as an ordinary consumer product (Banks et al. 2015; Hoek, Edwards, et al. 2022).

Each measure had a strong empirical, logical or theoretical foundation. Randomised controlled trials of very low nicotine cigarettes (VLNCs) found that people given VLNCs typically reduced how many cigarettes they smoke, made more quit attempts, and were more likely to quit successfully (Walker et al. 2012; Donny et al. 2015). Evidence reviews provided further support for VLNCs’ likely impact (Donny and White 2022; Hatsukami et al. 2022) and modelling predicted this measure would lead smoking rates to plummet (Ait Ouakrim et al. 2023).

Systematic reviews and meta analyses have found associations between exposure to tobacco retail outlets and increased risk of smoking uptake among young people who do not smoke (Finan et al. 2019; Marsh et al. 2021), and observational studies have reported increased relapse among people who live within close proximity of tobacco retail outlets (Pulakka et al. 2016; Chaiton et al. 2018). Although the smokefree generation policy had not been implemented and evaluated, it removed connotations of smoking as a ‘rite of passage’ and reframed tobacco as a highly toxic product (Berrick 2013); this measure had strong support from young people (Ball et al. 2023; Hoek, Lee, et al. 2022). Overall, all measures would have further denormalised smoking and helped ensure that, once prevalence had fallen, smoking could not resurge.

The Smokefree Action Plan, enacted as the Smokefree Environments and Regulated Products (New Zealand Government 2023) Amendment Act (SERPA) (Smokefree Environments and Regulated Products Act 2024), stimulated global discussion (Daube and Maddox 2021; McCall 2022). It focussed attention on measures to reduce tobacco’s addictiveness and easy availability, and posed new challenges to tobacco companies.

### Tobacco endgames, tobacco industry transformation, and repeal of the endgame measures

Endgame measures pose an existential threat to tobacco companies’ core business, yet align with their transformation narratives (Edwards, Hoek, Karreman, et al. 2022a). Philip Morris, for example, has outlined plans to ‘unsmoke the world’ (Philip Morris International, undated) while British American Tobacco (BAT) anticipates ‘a better tomorrow’ that does not include smoked tobacco (BAT 2020). Yet BAT and Imperial Brands Australasia (IBA), the two largest tobacco companies operating in Aotearoa, strongly opposed the legislation (BAT 2021; Imperial Brands Australasia 2022). Furthermore, Philip Morris contradicted their rhetoric by exploiting a loophole enabling them to offer a new budget-priced smoked tobacco product (Hoek, Ball, et al. 2022).

Covert behaviour, such as astroturfing (creating obstensibly authentic front groups to communicate industry arguments) also continued. Both BAT and IBA supported retailers’ concerns that the SERPA measures would reduce store revenue and overall business viability, despite research that questioned these claims (Robertson et al. 2018; Robertson and Marsh 2019). Specific astroturfing examples include a petition opposing proposals to reduce tobacco store numbers (Cheng 2021) and the ‘Save our Stores’ campaign, which also took the guise of a community-led initiative (Hancock 2023; Ozarka and Hoek 2023).

To the surprise and great disappointment of many, the coalition government formed in late 2023 announced it would repeal the endgame measures. The repeal evoked widespread outrage as numerous national and international groups campaigned to protect the SERPA measures. Despite widespread opposition from Māori and Pacific communities, medical and public health experts, young people and the wider public, the endgame measures were repealed in February 2024. This highly unpopular decision raised many questions about the tobacco industry’s influence on policy in Aotearoa.

Although media investigations had exposed tobacco companies’ role in fomenting opposition to the SERPA measures, it is more difficult to document exactly how these companies lobby politicians, even when known personal connections exist (Hoek, Edwards, et al. 2024b). Nonetheless, analysing politicians’ speeches and media statements allow us to investigate whether they used arguments advanced by tobacco companies. In the following sections, we analyse common industry claims and politicians’ use of these to justify the repeal. Evidence politicians used industry discourse, intentionally or not, would add to concerns that tobacco companies and their lobbyists had influenced the repeal decision.

Using a narrative analysis, we reviewed media reports and commentaries, interviews with politicians, and tobacco companies’ submissions on the Smokefree Action Plan and SERPA legislation and regulations; we then critically examined core industry arguments and explored their presence in coalition politicians’ discourse.

- RQ1: How did tobacco companies develop prohibition and public safety arguments when opposing the SERPA measures?
- RQ2: How did coalition government members adopt and use tobacco industry arguments?

### The Policy Dystopia Model

We used the Policy Dystopia Model (PDM) (Ulucanlar et al. 2016), an evidence-based conceptual model, as a framework for reviewing arguments used to explain and justify the repeal of the SERPA’s endgame measures. The PDM suggests tobacco companies follow a sequential strategy when faced with new public health policies (Ulucanlar et al. 2016). They first attempt to defeat proposals by moving these off the political agenda before policy design occurs; failing that, they attempt to delay the policy process and weaken the proposed measures. Other strategies include working to overturn the legislation, the eventual outcome in Aotearoa (NZ Government, 2024), and non-compliance, used when policy measures are implemented despite industry opposition.

The PDM also outlines arguments (or discursive strategies) that tobacco companies use to oppose policy. These include identifying unanticipated ‘costs’ that will affect ‘the economy, law enforcement, the law, politics and governance, and social justice’ (p. 21) (Ulucanlar et al. 2016). For example, tobacco companies may argue that introducing a policy will see legitimate trade decline (as retailers go out of business) and illicit trade increase, with a corresponding decline in excise tax revenue. Other claims imply policies represent excessive government intervention (the ‘nanny state’), reduce personal freedoms and autonomy, and create a ‘slippery slope’ that will see other ‘rights’ removed. This reasoning segues into arguments that underserving groups (particularly criminals) will benefit while the general public faces greater costs and threats to their wellbeing. Finally, tobacco companies dispute policies’ public health benefits and argue new measures will have unintended outcomes (e.g. the smokefree generation will position smoking as a forbidden fruit, thus making smoking more appealing to youth). Overall, these arguments predict public health policies will lead to unintended, harmful, and potentially catastrophic outcomes.

These arguments are not new to policy making in Aotearoa (Waa et al. 2017); based on earlier analyses, we examined two key PDM arguments: claims the SERPA measures unreasonably restricted important freedoms and amounted to prohibition, which often segues into a second PDM domain: threats to public safety, arguably attributable to rising illicit trade and the increased criminal activity it spawns.

### Industry arguments: prohibition and restricting freedoms

Tobacco companies often describe policies restricting their operations as prohibition or prohibitionist (Malone and Proctor 2022). These claims rely on a libertarian ideology that rejects measures limiting individual actions, even when there is strong evidence these limits could bring societal benefits (Schmidt 2022). Further, even an economic rationale, which views markets as self-regulating (and thus not in need of government regulation), overlooks the fact that addiction removes choice, which is fundamental to self-regulation. By focussing on short-term individual choice and market justice, prohibition claims shift attention away from smoking’s harms and citizens’ right to protection from toxic and addictive products. By privileging economic outcomes over population health, this argument reduces pressure on governments, which have a responsibility to safeguard citizens’ rights. These arguments also undermine Indigenous people’s sovereignty, given addiction is closely tied to colonisation (Waa, Maddox, et al. 2020).

Prohibition arguments featured in several submissions tobacco companies made on the Action Plan and SERPA legislation. Japan Tobacco International (JTI) claimed: ‘*Prohibition as demonstrated in the past, is ineffective in reducing the consumption of harmful products*’ and argued a ‘*dramatic reduction*’ in the nicotine content of cigarettes amounted to ‘*prohibition*’ that would lead to *‘dire consequences’* (Japan Tobacco International 2022). IBA argued: ‘*Mandating very low nicotine levels is equivalent to prohibiting the regular cigarettes currently consumed by millions of adult smokers’* (IBA 2022), and BAT described both denicotinisation and the smokefree generation policy as forms of prohibition (BAT 2021).

None of these companies provided compelling evidence to support their claims, and the prohibition argument fails on several grounds. First, cigarettes and nicotine products would have remained available, making prohibition a difficult argument to sustain. Second, while this reasoning tries to evoke US alcohol prohibition, bootlegging and non-compliance, this interpretation is overly simplistic, given alcohol prohibition was designed for a different product and located in a different setting and era. Despite its problems, US prohibition was not ineffective and alcohol consumption decreased (Blocker Jr 2006), with corresponding public health benefits. Third, precedents exist for ending use of highly toxic products, such as leaded petrol and paints. Fourth, the very high regret people who smoke experience, and the sustained efforts many make to quit smoking, do not support inferences they would reject the proposed measures (Fong et al. 2004; Hoek, Waa, et al. 2024; Wilson, Edwards, et al. 2009). Finally, denicotinisation has very strong support, including from people who reported voting for a coalition government party (Health Coalition Aotearoa 2023).

Nor did young people want retailers to remain able to sell them tobacco; a survey of youth and young adults found strong support for all SERPA measures (Ball et al. 2023). An in-depth study probing questions of addiction found young people understood freedom from addiction would require regulation and reported that most welcomed policies they thought would provide that protection (Hoek, Lee, et al. 2022).

Despite these logical and empirical flaws, coalition government members drew on the prohibition narrative. Associate Health Minister, Casey Costello, accused ‘*the last Government … * [of] *moving towards an untested regime with a focus on prohibition*’ (Hansard 2024), and mistakenly described the smokefree generation policy as ‘*prohibition of those born after January 2009 to buy cigarettes*’ (the policy ended sale of tobacco products to young people, a different proposition) (Hansard 2024). Health Select Committee chair and National MP, Sam Uffindell, described the measures as ‘*a prohibition style approach*’ (Hansard 2024).

### Industry arguments: illicit trade and comprehensive harms

Prohibition arguments often segued into claims about illicit trade, which tobacco companies argued would increase gang power and reduce public safety (BAT 2021). These assertions move attention away from smoking’s harms and suggest that risks to population health occur not via smoking’s harms but through unintended consequences caused by illicit trade.

Moving from a philosophical position to a concrete dystopic vision arguably aims to generate public anxiety and hostility to the policies. BAT’s submission on the Smokefree Action Plan claimed: ‘*Experience has demonstrated that prohibition does not work. It merely hands over control of the market to criminal organisations who would willingly supply illegal, unregulated products to people on the black market*’ (BAT 2021). IBA’s submission on the SERPA Bill went further, suggesting that: ‘*Prohibition fuels criminal supply networks that have no interest in product quality, no qualms about selling to children, and no desire to pay taxes. The growth of a black market can also create a gateway to other illicit substances, and fund networks involved in terrorism*’ (IBA 2022). JTI made similar claims, noting that ‘*The profits made from the illegal trade are also known to fund other activities such as terrorism and people trafficking which harm all of society*’ (Japan Tobacco International 2022).

References to ‘prohibition’ led into a dystopic narrative that connected the policies with rising global terrorism and aligned with the Government’s ‘cracking down on crime’ trope (Goldsmith and Mitchell 2024). Ironically, IBA felt concerned about ‘product quality’ yet apparently unmoved by the extreme harmfulness of their smoked tobacco products. Nor do concerns that tobacco may be sold to children sit logically alongside the tobacco industry’s long history of targeting children (Ling and Glantz 2002).

Tobacco companies provided no evidence to support their gloomy prognostications, which they had also used to oppose plain packaging and impede international policy progress (Waa et al. 2017; Lie et al. 2018), and their arguments again failed on several grounds. Analyses of discarded packs, which identify packs that do not comply with national packaging regulations (i.e. are likely to have been smuggled) found estimates of illicit tobacco in Aotearoa had remained stable over more than a decade (Bullen et al. 2023; 2024; Marshall et al. 2013; Wilson et al. 2022; Wilson, Thomson, et al. 2009). During this period, governments had introduced large excise tax increases and plain packaging, both of which tobacco companies had predicted would promote illicit trade (Waa et al. 2017). Consumption gap analyses, which compare the volume of tobacco released for sale and self-reported tobacco consumption, and attribute any gap to illegally imported tobacco, also found illicit tobacco had declined (Bullen et al. 2023; 2024). Even an industry funded report estimated that the volume of illicit tobacco consumed between 2019 and 2022 had fallen from 230 to 167 million kg (KPMG 2023).

Nor did crime statistics support claims that public safety would decrease. Police data showed ram-raids (use of cars to break into retail outlets) had decreased substantially after 2022 (Chambers 2022), presumably following government funding to enhance store security. Furthermore, analyses of items stolen during ram raids revealed that cash and cash registers were the most frequently stolen items in ram raids. Although nearly a quarter of ram raids (24%) targeted cigarettes and tobacco, only 14% of all ram raids successfully stole these items and, overall, three-quarters of ram raids during the 2020–2021 period examined did not involve tobacco products (Hoek et al. 2024a).

Claims implying that tobacco thefts would increase and fuel the illicit market failed to consider the rapid declines in smoking prevalence predicted to follow denicotinisation (Ait Ouakrim et al. 2023). Because denicotinised tobacco would be non-addictive and easier to quit, demand would decrease and illicit supply would become logically less lucrative (Hoek, Lee, et al. 2022). Further, requiring tobacco outlets to meet high security standards (among other criteria) would reduce stores’ vulnerability to ram raids and thus the volume of stolen product available for illegal sales.

Research studies suggest tobacco companies’ public safety arguments amounted to disinformation (Ozarka and Hoek 2023), and the Ministry of Health also explained errors in tobacco companies’ claims (Newton 2024a). However, Government Ministers often drew uncritically on industry rhetoric. The Prime Minister claimed the SERPA policies would create ‘* … an increased black market - an untaxed black market - for* [cigarettes]’ (RNZ 2023), and explained ‘*We think it* [the new policies] *will encourage a black market, we think it will encourage more crime, and as a result we're sticking with the status quo*’ (Palmer 2023).

Prior to his appointment as Health Minister, Dr Shane Reti had supported denicotinisation and attempted to introduce this measure more quickly than originally planned (Reti 2022). However, post-election, he noted concerns about rising crime (Checkpoint 2023). When pressed for evidence, he relied not on peer-reviewed independent data, but on anecdotes from retailers, the group tobacco companies had used as a façade in their astroturfing activities. Dr Reti noted: ‘*And their* [retailers’] *very clear indication that they are deeply concerned that they could be at risk of increased crime, with a reduction from the 6000 distributing networks down to 600 and so that has been a concern*’ (Checkpoint 2023).

Overall, whether intentional or not, coalition government MPs regularly cited tobacco industry arguments when defending the repeal decision. None appeared to have considered either the (lack of) logic or evidence underpinning the claims they rehearsed or how the repeal would affect population wellbeing.

### Compliance with the World Health Organization Framework Convention on Tobacco Control

Tobacco companies have an overwhelming commercial interest in opposing effective tobacco control policies and a long history of disrupting policies designed to regulate their harmful products (World Health Organization 2009). They have made misleading or false assertions (Waa et al. 2017), lobbied and influenced decision-makers (Rotman et al. 2022), manipulated research ‘evidence’ (Bero 2005), undermined independent researchers (Matthes, Alebshehy, et al. 2023a), and developed alliances by creating or cultivating front-groups to promote their goals (Matthes, Kumar, et al. 2023b). Nor are these activities simply historical; recent studies report on their sustained efforts to disrupt new policy measures (Matthes et al. 2023).

The Framework Convention on Tobacco Control (FCTC), developed under the auspices of the World Health Organization, is a global treaty that responds to the tobacco epidemic (World Health Organization 2003). The FCTC explicitly calls on signatories to protect policy making from tobacco industry influences; Article 5.3 states that: ‘*in setting and implementing their public health policies with respect to tobacco control, Parties shall act to protect these policies from commercial and other vested interests of the tobacco industry in accordance with national law’* (World Health Organization 2003). Further, Article 5.3 requires governments to interact with tobacco companies only as required for regulatory purposes, and states that all interactions must be documented and transparent (World Health Organization 2013).

Reports that Minister Shane Jones had taken ‘soundings’ from tobacco companies intensified concerns (Vance 2024), which were not allayed by reports that he did not intend to give the FCTC ‘* … one iota of attention*’ (Newton 2024b). Casey Costello, the Associate Health Minister responsible for leading repeal of the endgame measures, has also come under close scrutiny because of her former role with a ‘think tank’ that receives tobacco industry funding (Hoek, Edwards, et al. 2024b) and failure to follow advice from her Ministry (Hoek, Ball, et al. 2024a). News articles have challenged her inability to explain who wrote a mystery document forwarded from her office to the Ministry of Health and the Associate Minister’s sustained failure to explain this document’s provenance has raised concerns it was written by a tobacco company staff member (Espiner 2024). This document, which she released in a heavily redacted form when required by the Ombudsman, had previously been leaked to a journalist and is now publicly available (Unknown 2024). It proposed measures that align with tobacco companies’ calls for more liberal regulation of non-combusted nicotine products (e.g. allowing oral nicotine products to be sold) and suggested reducing excise tax on non-combustible products. The Associate Health Minister has signalled she plans to expand the nicotine product market place and, in mid-2024, she reduced the excise tax on heated tobacco products; these actions have fuelled concerns that tobacco companies are shaping smokefree policy. Overall, Mrs Costello’s failure to follow evidence-based advice from the Ministry of Health has inevitably raised questions about how the smokefree policy agenda had been determined. Her behaviour has strengthened calls for greater transparency regarding the tobacco industry’s interactions with people working in all parts of a policy cycle and reinvigorated interest in more robust lobbying regulation.

### Regulating lobbying

Definitions of lobbying vary, though the Organization for Economic Co-operation and Development (OECD) defines it as ‘the act of lawfully attempting to influence the design, implementation, execution and evaluation of public policies and regulations administered by executive, legislative or judicial public officials at the local, regional or national level’ (OECD 2021). Aotearoa currently requires disclosure of all meetings with tobacco industry representatives; the Ministry of Health website provides details of staff engagement (Ministry of Health 2024). Ministers’ diaries contain information about past appointments but do not list encounters that occur at events or other social settings and so may not include informal interactions with lobbyists. Furthermore, while diaries provide information about Ministers’ meetings, they do not detail meetings between Ministers’ staff and industry representatives. Information about these engagements may be obtained under Aotearoa’s primary freedom of information law, the Official Information Act 1982 (OIA). However, the OIA has limited ability to capture lobbying activities (Williams 2014) and reviews have questioned how effectively agencies respond to requests (Price 2005).

More complete disclosure provisions would require lobbyists to provide information about their activities, including who commissioned them, the funding they received, details of who they lobbied and the issue on which they focussed (OECD 2021). Disclosures should cover Ministers and their staff, as well as MPs, given MPs play key roles in policymaking (e.g. they propose Members’ bills and scrutinise the Executive in the House). Yet in Aotearoa, lobbying of MPs has little, if any, public oversight and comments made by Minister Shane Jones appear to suggest that lobbying by tobacco companies may have occurred even prior to his election (Newton 2024b).

Unlike Australia, where third-party lobbyists must register their details on a national database, Aotearoa has no register of lobbyists. Even in Australia, legal experts have raised concerns that current requirements capture only around 20 percent of active lobbyists (i.e. those practising as in-house lobbyists are not required to register) and called for a more comprehensive system (Ng 2021). They have also argued that lobbyists should provide more information, including details of which officials they contact, at what times, and concerning which policy questions (Ng 2021).

Aotearoa also lacks a code of conduct for lobbyists. Although the Ministry of Justice has attempted to develop a voluntary code of conduct, their draft attracted criticism, with one expert reportedly describing it as ‘essentially irrelevant’ because it failed to provide any consequences for breaches of the code (The Law Association 2024). Aotearoa now appears a case study documenting the risks of a laissez-fare approach to lobbyists. Experts have called for a law to regulate lobbying, introduce a public register of lobbying, require stand-down periods that apply to former public officials, outline a code of conduct, and a establish regulatory body to oversee these mechanisms (p.8) (Rashbrooke 2024).

Without this structure, lobbying will occur in the shadows and privilege commercial determinants of health that have undermined and pre-empted public health policy, and entrenched health inequities (Yasbek 2024). In Aotearoa, the absence of lobbying regulation effectively enables tobacco companies to obstruct policy. While circumstantial, the presence of a mystery document advancing ideas that would benefit tobacco companies, and the adoption of some proposed actions, has raised concerns. Furthermore, similarities between claims politicians made to justify the repeal and tobacco industry arguments, has added to concerns that tobacco companies successfully lobbied to overturn the three key SERPA measures.

### Conclusions and recommendations

The repeal leaves Aotearoa without a robust plan to achieve the Smokefree 2025 Goal, reduce inequities and greatly decrease the many preventable deaths caused by smoking. The replacement ‘plan’ announced in late 2024 returns to individually-focussed measures known to work slowly and inequitably; it lacks courage, ambition and foresight (Waa, Ball et al. 2024). Given the goal must be realised in 2025, we recommend reversing the repeal and following former Health Minister Dr Reti’s proposal to introduce denicotinisation rapidly and ahead of any other measure (Reti 2022).

Unrestricted lobbying by people representing the tobacco industry (or other harmful industries) poses a serious threat to public health policies and raises questions about influences on coalition government members’ judgment. We thus also recommend introducing lobbying regulations that promote transparency, privilege disclosure over confidentiality, and protect public interests over corporate profits. Finally, we recommend an independent inquiry into tobacco control policy-making, tobacco industry lobbying and influence on policy, including ascertaining the source of Mrs Costello’s influential mystery document. Reconvening key members of the original MASC Inquiry to undertake this investigation could foster confidence in the review, enable active participation by civil society, and protect the views and aspirations of Māori.

Our analyses have limitations that future research could address. For example, we focussed on two of the PDM’s arguments and future work could examine economic arguments (e.g. loss of Government revenue) as well as narratives used to reject interventions (e.g. the policy will not work or is not necessary). More detailed analyses of other narratives could create a comprehensive framework of tobacco industry interference. We predict this additional work would reiterate the need for endgame measures to address health inequities caused by smoking, including deaths from cancer, and reassert realising the Tupeka Kore goal as a crucial priority for population wellbeing.
